# Pleomorphic Invasive Ductal Carcinoma of the Breast in a Patient with Huntington's Disease

**DOI:** 10.1155/2014/979137

**Published:** 2014-12-09

**Authors:** Sami Shousha

**Affiliations:** Department of Histopathology, Imperial College Healthcare NHS Trust and Imperial College, Charing Cross Hospital, Fulham Palace Road, London W6 8RF, UK

## Abstract

A pleomorphic invasive ductal carcinoma developed in a patient with Huntington's disease. The tumour showed marked nuclear pleomorphism and contained large number of bizarre tumour giant cells and abundant abnormal mitoses. Tumour cells showed nuclear vesicles and inclusions similar to those described in nuclei of neural cells in patients with Huntington's disease. The case suggests that, in some patients, tumour morphology may reflect specific individual features.

## 1. Introduction

Huntington's disease is an autosomal-dominant progressive neurodegenerative disorder characterised by chorea, dystonia, and cognitive decline [1]. Pathologically there is neurodegeneration of the basal ganglia and cerebral cortex [2] and characteristic nuclear and cytoplasmic inclusions that contain mutant huntingtin and polyglutamine [1]. The nuclear inclusions have been described in neurons as well as in peripheral tissues and can be single or multiple [3]. The gene for the disease is located on the short arm of chromosome 4 and it encodes the production of “huntingtin protein” which is expressed in neurons as well as in many other tissues and organs. Huntingtin is thought to be involved in transcriptional events, protein trafficking, and vesicle transport [4]. It is present in both the cytoplasm and the nucleus [5]. The mutant gene results from expanded CAG repeat leading to a polyglutamine strand of variable length at the N-terminus. This results in the production of a mutant huntingtin protein, which is ubiquitously expressed throughout the body in patients with the disease [2]. This affects biological processes in all cells [5] and results in abnormalities in peripheral tissues which are thus considered to be related to the presence of the mutant protein rather than being secondary to the neurodegenerative changes [2]. Although patients with Huntington's disease have a reduced risk of developing cancer in general [6, 7], with an overall risk of 0.54 for breast cancer [7], it seems that when cancers develop in these patients they show enhanced progression [8]. Huntingtin has been demonstrated in the nuclei of normal and neoplastic breast epithelium [8]. In experimental animals, mutant huntingtin was shown to accelerate tumorigenesis, increase epithelial-mesenchymal transition of cancer cells, and favour lung metastasis [8]. Breast carcinomas developing in animals bearing the mutant Huntington gene are bigger and less differentiated and show higher expression of Ki67 protein, compared to tumours arising in animals with the wild gene [8]. The former tumours also expressed alpha smooth muscle actin and vimentin, increased HER2 membrane staining, and decreased levels of E-cadherin and beta-catenin [8].

I here present a case of breast carcinoma developing in a patient with Huntington's disease. The tumour was of the uncommon pleomorphic invasive ductal type and showed unusual microscopic features which probably reflect the patient's polyglutamine disease.

## 2. Case Report

The patient presented with a 5 cm mass in the left breast. The patient had a long standing history of Huntington's disease. A breast core biopsy showed a poorly differentiated carcinoma which proved to be negative for oestrogen and progesterone receptors as well as for HER2 and cytokeratin 5 but positive for cytokeratin 7. No DCIS elements were present. The tumour was so undifferentiated that it warranted considering the possibility of a metastasis. Further immunohistochemistry showed that the tumour was negative for TTF1 and WT1, and clinical investigations failed to demonstrate any other tumours. The tumour was considered to be a primary grade 3 invasive ductal carcinoma of the breast, and mastectomy with axillary lymph node clearance was recommended.

**Figure 1:**
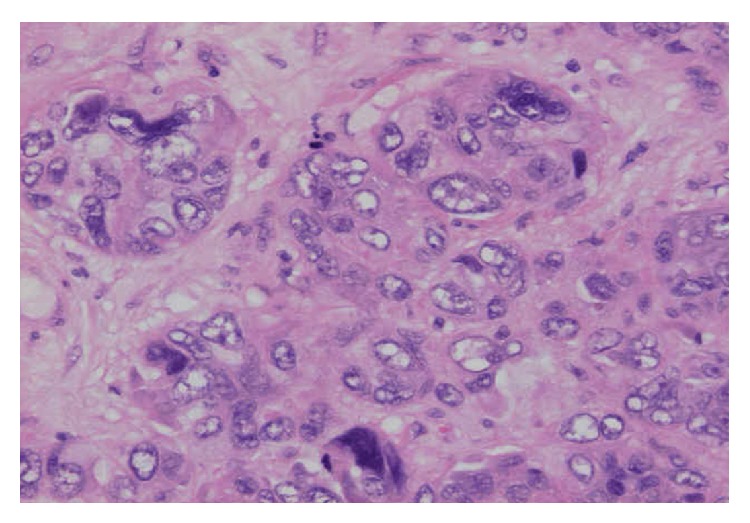
Tumour cells with unusual shapes and variable sizes including multinucleated giant cells. Note the pleomorphic vesicular nuclei and abundant pale stained cytoplasm (haematoxylin and eosin ×200).

**Figure 2:**
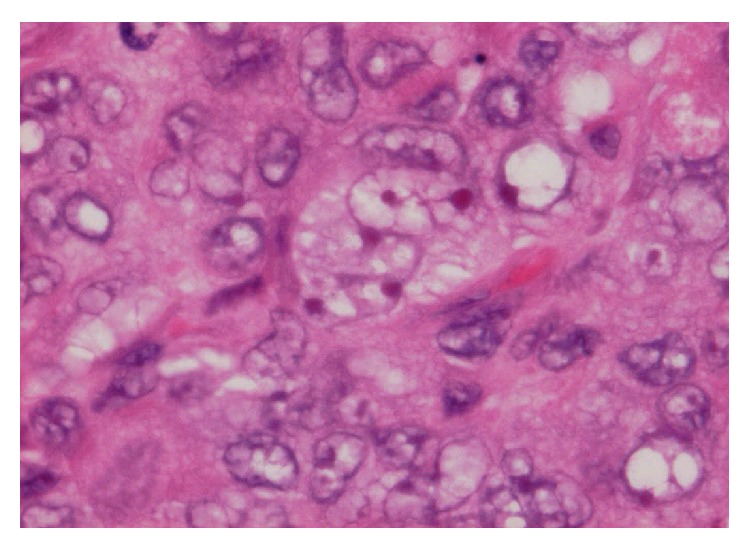
Mono- and multinucleated tumour cells. Note the presence of nuclear vesicles and pink stained inclusions (haematoxylin and eosin ×400).

**Figure 3:**
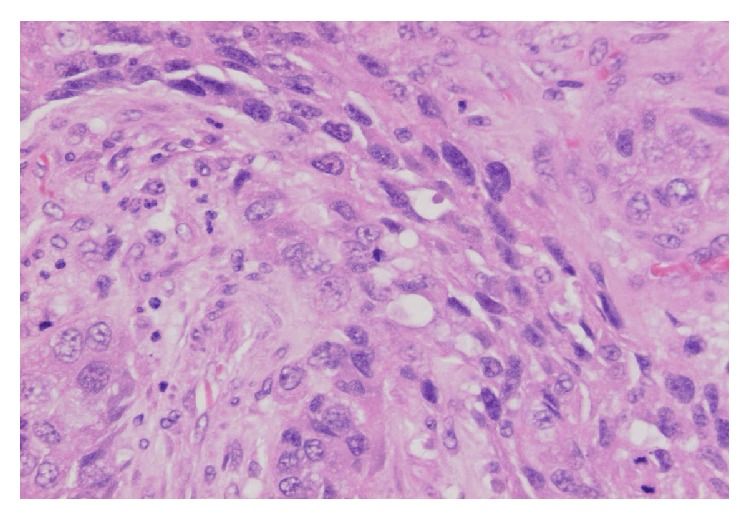
An area with spindle-shaped tumour cells (haematoxylin and eosin ×200).

**Figure 4:**
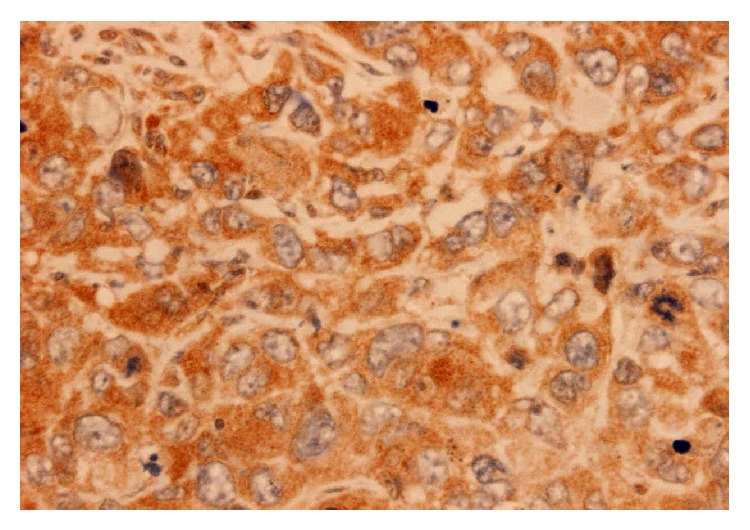
Positive cytoplasmic granular staining of tumour cells using anti-HD antibody.

The mastectomy specimen showed a fairly well defined hard tumour mass, 48 mm in maximum dimension that appeared completely excised. Microscopically, the tumour showed marked nuclear pleomorphism involving more than 50% of tumour cells as well as scattered large numbers of multinucleated tumour cells, features which are consistent with pleomorphic invasive ductal carcinoma [9–11]. The tumour cells varied widely in size and shape with some having bizarre features with diffuse homogeneous pale stained cytoplasm and markedly enlarged pleomorphic nuclei with prominent nucleoli (Figure 1). Abundant vesicles were present in the nuclei with some also showing bright red inclusions (Figure 2). Numerous huge multinucleated giant tumour cells were scattered throughout the tumour, having the same type of nuclei as the mononuclear tumour cells (Figure 1). Abundant mitotic figures were present, many showing abnormal patterns. In some areas streams of neoplastic spindle-shaped cells were present (Figure 3). Immunohistochemistry showed that the tumour cells were strongly positive for cytokeratin 7, p53, and E-cadherin, focally positive for S100 and CD68, and weakly positive for EGFR, features which are all consistent with the diagnosis of pleomorphic invasive ductal carcinoma [9–11]. An immunoperoxidase stain for huntingtin protein using the monoclonal antibody anti-HD (81-190) mab (Abnova (Taiwan) corporation, 1/100) showed diffuse cytoplasmic granular staining in most tumour cells (Figure 4). All 19 lymph nodes dissected from the axilla were free of tumour.

Because of the patient's general health, no further treatment was given. The patient died 15 months later from “complications of Huntington's disease.”

## 3. Comment

Although many of the features seen in this case are similar to those described in pleomorphic invasive ductal carcinoma, there are additional features including peculiar cytoplasmic and nuclear changes that are more akin to those described in neural cells of patients with Huntington's disease. This raises the possibility that, in some patients, tumour morphology may reflect specific personal features, leading to a form of “individualised tumour morphology.” The significance of the positive cytoplasmic staining obtained with the huntingtin antibody used is not clear, as it is possible that the antibody may react with the normal as well as the abnormal protein which is known to be ubiquitous. Examination of more tissues is needed to reach a conclusion in that respect.
